# Clinical Action against Drunk Driving

**DOI:** 10.1371/journal.pmed.1002231

**Published:** 2017-02-14

**Authors:** Donald A. Redelmeier, Allan S. Detsky

**Affiliations:** 1 Department of Medicine, University of Toronto, Toronto, Ontario, Canada; 2 Evaluative Clinical Sciences Program, Sunnybrook Research Institute, Toronto, Ontario, Canada; 3 Institute for Clinical Evaluative Sciences, Toronto, Ontario, Canada; 4 Division of General Internal Medicine, Sunnybrook Health Sciences Centre, Toronto, Ontario, Canada; 5 Center for Leading Injury Prevention Practice Education & Research, Toronto, Ontario, Canada; 6 Institute for Health Policy Management and Evaluation, University of Toronto, Toronto, Ontario, Canada; 7 Department of Medicine, Mount Sinai Hospital and University Health Network, Toronto, Ontario, Canada

## Abstract

In advance of a safety campaign on 17 March 2017, Donald Redelmeier and Allan Detsky call on physicians and clinical colleagues to reduce the chances that patients will drive drunk.

In 2014, over 100,000 people in the United States were hospitalized because of alcohol-related traffic crashes, and 9,967 died (exceeding the 6,721 US deaths from HIV in the same year) [1,2]. On 17 March 2017, the National Highway Traffic Safety Administration (NHTSA) plans to promote a safety campaign against drunk driving. Past estimates suggest that law enforcement against drunk driving reduces traffic fatalities by 20% and that high-probability detection is more effective than high-severity punishment [3,4]. Yet, 12 states in the US, including the large states of Texas and Minnesota, prohibit random sobriety checkpoints, and the remaining have uneven efforts against drunk driving [5]. This Perspective identifies some groups with a vested interest in preventing drunk driving, describes reasons for the relative inaction, and proposes more action by physicians.

Traditionally, physicians and allied health care providers have deferred to others about how to address the health risks of drunk driving. One explanation is that drunk driving is a behavioral choice, and behavioral change is difficult to effect in a time-limited clinical encounter [6]. Moreover, preventive care may provide less evident benefit to the patient than prescribing an acid blocker, for example, to treat symptomatic alcohol-induced gastritis. While a pregnant woman who drinks alcohol is likely to be warned by her obstetrician or midwife on the risks to fetal development, most patients in our experience who are prone to drunk driving are easily missed because physicians rarely ask about drunk driving, despite often asking about alcohol. As a consequence, standard care may fail to identify this prevalent, modifiable, and serious health risk.

Vehicle manufacturers are the most powerful commercial group that can promote traffic safety. Over time, this industry has carefully developed and marketed technologies to protect drivers, such as seat belts, airbags, antilock brakes, and safety glass. Currently, the main technology to prevent drunk driving is an ignition interlock that forces drivers to have a breath test before engine engagement. This device, now imposed only on the vehicles of convicted drunk drivers, is unlikely to be adopted broadly any time soon unless manufacturers want to boast that they make the safest cars for those prone to drunk driving. The net result is that vehicle regulators in the US are unable to rely on manufacturer innovations or economic forces to prevent drunk driving.

Other large groups have even less incentive to promote sobriety while driving. Alcohol manufacturers promote “responsible drinking,” which is a vacuous tautology because adverse events can be deemed “irresponsible” by rhetorical hindsight. Celebrities in the entertainment industry are occasionally charged with drunk driving yet rarely express enduring regret. Lawyers gain little financial benefit from deterring drunk drivers, whereas some profit substantially by defending those who have deep pockets and are charged with drunk driving. Individual police officers themselves sometimes consider traffic enforcement as low-prestige work with little career satisfaction [7]. Driving enthusiasts argue that enforcement mostly inconveniences safe drivers to catch a few deviants. Those caught driving drunk are rarely grateful for the penalties.

Sometimes medical science can inspire behavior change, and drunk driving could seem amenable to research because of the large number of incidents. A rigorous clinical trial, however, cannot be conducted unless broad regions are willing to implement interventions in a thorough manner. An epidemiological analysis contrasting different states would also be easily misinterpreted because of dissimilarities across regions and diversity within regions (for example, the risk of dying in an alcohol-related traffic crash is three times higher in South Carolina than in New Jersey even though both states allow random sobriety checkpoints) [1]. Because individuals are not randomly assigned to driving locations, furthermore, the confounders are almost boundless [8]. These research limitations mean that scientific evidence is unlikely to cause people to stop drunk driving [9].

## Activism and Politics

The most prominent body advocating change has been Mothers Against Drunk Driving (MADD), a citizen group with deeply motivated members [10]. The mission of MADD is to “stop drunk driving, support the victims of this violent crime, and prevent underage drinking.” MADD has successfully pushed for drunk-driving laws, increased public awareness, designated-driver initiatives, alcohol ignition interlock programs, and victim impact panels. Yet, MADD is mostly a volunteer organization, with fewer than 500 employees. MADD has also been criticized for having administrative costs and for shifting towards broader prohibitions against alcohol consumption [11]. Ultimately, MADD has no power to enforce drunk-driving laws.

**Fig 1:**
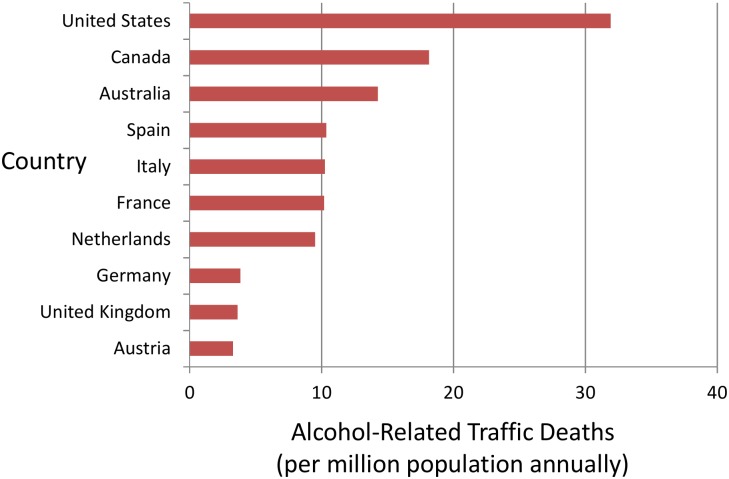
Alcohol-related traffic fatalities in the US and other countries. Histogram of alcohol-related traffic fatalities in the US and other countries. The vertical axis shows ten countries sequenced by death rates. The horizontal axis shows alcohol-related traffic deaths as fatalities per million population annually. Data are from the Organisation for Economic Co-operation and Development (OECD) Road Safety Annual Report 2015 authored by the International Traffic Safety Data and Analysis Group (IRTAD) and available at the following website: http://www.oecd-ilibrary.org/transport/road-safety-annual-report-2015_irtad-2015-en. The death rates were calculated from Table 1.3 of the report and individual country alcohol profiles. The results show high rates of alcohol-related traffic fatalities in the US relative to other countries.

Politicians in the US seldom discuss traffic safety with the same zeal that they direct at debates on economic growth, domestic terrorism, public scandals, gun deaths, climate change, and other public-policy priorities. Indeed, making a political issue of drunk driving can carry particular risks because the historical failure of prohibition decades ago (1920 to 1933) means that a well-intentioned politician is easily ridiculed or mischaracterized as being antialcohol [11]. A US politician who seeks re-election will rarely promise action against drunk driving. The US political process, therefore, exchanges safety for freedom and tolerates a remarkably high rate of alcohol-related traffic fatalities relative to other countries (Fig 1).

## Medical Initiatives

Health care providers are perhaps the one remaining large powerful group with a profound commitment to health. Physicians and allied life-saving professionals sometimes advocate to reduce cigarette smoking, drug abuse, domestic violence, or other societal epidemics. Drunk driving causes major mortality and morbidity that is utterly preventable, unlike many advanced diseases. The losses are also tragic because offenders usually have no malicious intent yet many lives are irrevocably altered (including their own). Because the market forces for commercial industries run in a different direction, physicians could advocate more for what works against drunk driving (Box 1) [12]. Civic advocacy, however, rarely leads to immediate gratification and sometimes deteriorates into dissenting backlash [13].

**Box 1. Physician Strategies to Prevent Drunk Driving**

- Alcohol screening and brief interventions for patients with alcohol problems
- Physician warnings for patients who sometimes drink and drive
- Treatment of patients diagnosed with alcohol dependence
- Counseling of patients not to ride with drunk drivers
- Supporting enforcement of laws against drunk driving
- Promoting sobriety checkpoints in local communities
- Lending voice to mass media campaigns against drunk driving
- Joining multicomponent interventions in coalitions of community group members

Patients do not want to become traffic statistics, tend to listen to their physicians, and take advice seriously. When asked about alcohol consumption, for example, patients often respond truthfully inside a private medical relationship. A simple extension, therefore, might be for physicians to also ask patients about past episodes of drinking and driving. Patients identified, in turn, could be recommended a taxi service, ride-sharing option (e.g., Uber and Lyft), or a designated-driver substitute. The intent is to suggest safer alternatives so patients who drink do not need to drive [14]. The intent is not to preach sobriety or to betray patient trust. Ideally, these harm-reduction strategies should be planned in advance because later inebriation will predictably impair judgment.

In Canada, recent financial incentives have been effective at motivating physicians’ warnings for medically unfit drivers and reducing the risk of a traffic crash for patients diagnosed with alcoholism [15]. A direct incentive for physicians’ warnings against drunk driving could be considered to address the problem in the US (the fee in Canada is C$36.25). Physicians need to first realize, of course, that an average drunk driver has a 5%–15% lifetime risk of dying in a traffic crash, physician warnings lead to a one-third relative reduction in the subsequent risk of a serious traffic crash, and most adults who drink and drive visit a physician in the year before dying [16]. The epidemic of drunk driving needs to be addressed in the US, and 17 March 2017 is a time for physicians to think more about clinical action against drunk driving.

## Abbreviations

IRTAD: International Traffic Safety Data and Analysis Group
MADD: Mothers Against Drunk Driving
NHTSA: National Highway Traffic Safety Administration
OECD: Organisation for Economic Co-operation and Development
